# Mean platelet volume change (∆MPV) and red blood cell distribution width (RDW) as promising markers of community-acquired pneumonia (CAP) outcome

**DOI:** 10.1186/s43168-020-00024-z

**Published:** 2020-08-20

**Authors:** Sahar Farghly, Randa Abd-Elkader, Randa A. El Zohne, Doaa M. Abd El-Kareem

**Affiliations:** 1 Department of Chest Diseases, Faculty of Medicine, Assiut University, Assiut, Egypt; 2 Department of Clinical Pathology, Faculty of Medicine, Assiut University, Assiut, Egypt

**Keywords:** Community-acquired pneumonia, Red cell distribution width, Mean platelet volume

## Abstract

**Background:**

Prognostic markers play an essential role in the proper management of community-acquired pneumonia. This research work aimed to evaluate the association of RDW and /or MPV with mortality and morbidity in patients with CAP to improve the yield of already used prognostic scores.

**Results:**

The current study enrolled 153 patients with community-acquired pneumonia (CAP). Out of them, 101 (64%) patients improved while 52 (36%) died. It was noticed that each of delta MPV and RDW (*P* < 0.001) had positive significant correlation with PSI and CURB-65. Delta MPV and RDW was significantly higher in patients who died (2.61 ± 1.01 vs. 1.78 ± 0.76; *P* = 0.01 for delta MPV and 16.50 ± 3.54 vs. 15.50 ± 2.81; *P* = 0.02 for RDW).

**Conclusion:**

Initial RDW and rising MPV during hospitalization for CAP is associated with more severe clinical characteristics and high mortality. Moreover, the use of RDW and delta MPV in patients admitted with CAP can improve the performance of prognostic scales.

## Background

Community-acquired pneumonia (CAP) is the fourth leading cause of death all over the world and plays an important role of morbidity and mortality [1–3]. Scoring systems have an essential role in the management of patients with CAP, and currently, there are several severity scores in use such as pneumonia severity index (PSI), CURB-65. However, these severity scores have some limitations and variations. For example, the CURB-65 and CRB-65 are crude scores for rapid assessment of the high-risk patients while PSI is believed to be useful for identifying low-risk patients Therefore, there is effort to improve the prognostic value of these severity scores.

Several biomarkers have been checked and verified for use in CAP which could improve the prognostic performance of severity scores [4, 5]. However, some of these biomarkers are non-specific and not sensitive. Others are expensive and are not always available immediately. Mean platelet volume (MPV) is done as a routine laboratory test that is measured in complete blood count, and it is considered a marker of platelet function and activation [6, 7]. A single elevated MPV measurement has been found to be associated with increased morbidity and mortality in various patient populations [8, 9]. Patients hospitalized with community-acquired pneumonia (CAP) are found to be at an increased risk of death in the hospital and following discharge [10–12]. The prognostic significance of MPV has been studied in only two small studies on CAP patients which were based on single MPV determinations [13, 14]. The clinical characteristics and prognosis of time-dependent MPV changes have not been investigated in the CAP population. We hypothesized that the MPV may reflect platelet activity and may be associated with an impaired host response. According to this hypothesis, an increasing MPV may be associated with poor outcomes, and may predict in-hospital mortality in ICU patients with severe pneumonia. To test our hypothesis, we examined MPV alterations in patients with severe pneumonia who had been admitted or transferred to the ICU

Red cell distribution width (RDW) is defined as a coefficient of variation of circulating red cells. It is affected by changes of red cell volume and is measured in the routine complete blood count (CBC). Few years early, RDW used in the clinical practice to diagnose different types of anemia; moreover, elevated RDW had a prognostic role in the outcome of some diseases, like cardiovascular disease, rheumatoid arthritis, colon cancer, and metabolic syndrome [15, 16]. Furthermore, few researches have reported RDW as a prognostic predictor of mortality in different populations [17]. The exact mechanism of variation in RDW is still unknown, but it is mostly associated with the process of oxidative stress and inflammation which reflects the prognostic role of RDW [17]. To our knowledge, RDW use does not imply any additional cost because it is routinely provided as part of the whole blood count by hemocytometry.

Several studies support the hypothesis that RDW may be a useful parameter for gathering either diagnostic or prognostic information on a variety of cardiovascular and thrombotic disorders [18, 19], although the link between RDW and cardiovascular disease is unclear [20]. In recent years, RDW has been associated with CAP outcomes, especially with 30-day and 90-day mortality and complicated hospitalization [21, 22]. This research work aimed to validate the role of RDW and MPV as promising markers of mortality and morbidity in patients with CAP to improve the yield of already used severity scores.

## Methods

This prospective study included 153 adult (> 18 years of age) patients admitted to Chest Department and RICU of Assiut University Hospital with a diagnosis of CAP between October 2017 and October 2019. Patients were prospectively recruited within 24 h of their arrival. CAP was defined as an acute disease with a radiological infiltrate that was not previously present and not due to another known cause and was associated with symptoms of lower respiratory tract infection (1). Exclusion criteria were (1) severe immunodepression (HIV infection or severe hematological diseases); (2) immunosuppressive therapy: prednisone or equivalent dose of > 20 mg daily for > 2 weeks, or any immunosuppressive regime therapy (azathioprine, cyclosporine, cyclophosphamide, and/or other immunosuppressant drugs); (3) leukopenia (< 1000 leukocyte per mm^3^) or neutropenia (< 500 neutrophils per mm^3^) and/or chemotherapy in the previous year; (4) pulmonary abscess (radiological cavitation), aspiration pneumonia, and obstructive pneumonia; (5) possible nosocomial origin (< 30 days from hospital discharge); and (6) known active neoplasia. All patients were followed up during their hospital stay, and those with a definitive diagnosis other than CAP were excluded. All of the patients were followed up until being discharged. Our study primary outcome variable was in-hospital mortality of patients with CAP. The secondary outcome variables were length of hospital stay, intensive care unit (ICU) admission, and mechanical ventilator requirement. This study protocol was approved by the Local Ethics Committee, and informed consent was obtained from all patients or next of kin.

### Data collection

Age, sex, tobacco and alcohol consumption, comorbid conditions (diabetes, liver, chronic kidney, hearth, and cerebrovascular diseases, non-active neoplasia, bronchiectasis, and previous CAP), and previous therapies (inhaled or oral corticoids and antibiotics) were recorded on admission. The following data were also recorded: days of duration of disease; CAP signs and symptoms (fever, cough, sputum, dyspnea, pleural pain) vital constants at the ED (temperature, respiratory and heart rates, arterial pressure, and oxygen saturation); analytic data (pH, PaO2, urea, Na, glucose, hematocrit, hemoglobin, red cell distribution width (RDW), and mean platelet volume (MPV); number of lobes involved and type of radiological condensation (alveolar, interstitial, or mixed); and complications (respiratory, cardiologic, renal, neurologic, digestive, and others), non-invasive mechanical ventilation (NIMV), need of ICU, and invasive mechanical ventilation (IMV). PSI and CURB-65 scores were calculated for all patients. All patients were admitted to the hospital for ≥ 24 h.

### Sample collection and processing

Blood was collected from anticubital fossa by experienced phlebotomists using a standardized atraumatic protocol using clean venipuncture and minimum tourniquet pressure. Needles used were 19–21 gauge. Specimens were maintained at room temperature (20–258 °C) not placed on ice, refrigerator, or water bath. Tubes kept capped upright at room temperature not exposed to vibration excessive mixing or agitation. Specimens containing any evidence of clotting were discarded. Two samples of 2 ml of venous blood in standard tubes contain ethylenediamine tetraacetic acid) EDTA) anticoagulant for complete blood count (CBC). The first one at the time of admission, and the second one after 5 days of admission. The CBC sample was examined within 1 h as recommended by BCSH guideline 2011 [23] to avoid bias due to excessive platelet swelling and to minimize variation due to sample aging. MPV and other blood count parameters were measured by an automated analyzer (ADVIA 2120i, Jermany) with LH 750 control system. In our laboratory, the range of normal MPV values is 7.6–8.6 FL. For analysis of time-dependent MPV changes, patients were categorized according to ΔMPV (MPV on discharge minus MPV on admission) into patients with no rising MPV (ΔMPV < 0.6 FL) and patients with rising MPV (ΔMPV ≥ 0.6 FL). RDW was reported as a part of the CBC results. RDW is the standard deviation of MCV and was measured in as percentage. A single RDW had been recorded from CBC of patients on admission. In our laboratory, the range of normal RDW values is 11:14%.

### Statistical analysis

Data was collected and analyzed those using SPSS (Statistical Package for the Social Science, version 20, IBM, and Armonk, NY). Continuous data was expressed in form of mean ± SD or median (range) while nominal data was expressed in form of frequency (percentage). Independent risk factors of mortality had been determined by multivariate regression analysis.

Receiver operator curve was used to determine cut-off of RDW and delta MPV for prediction of in-hospital mortality in patient with community-acquired pneumonia. Pearson correlation was used to determine correlation between PSI and CURB-65 with RDW and delta MPV. Level of confidence was kept at 95%; hence, *P* value was significant if < 0.05.

## Results

**Fig. 1:**
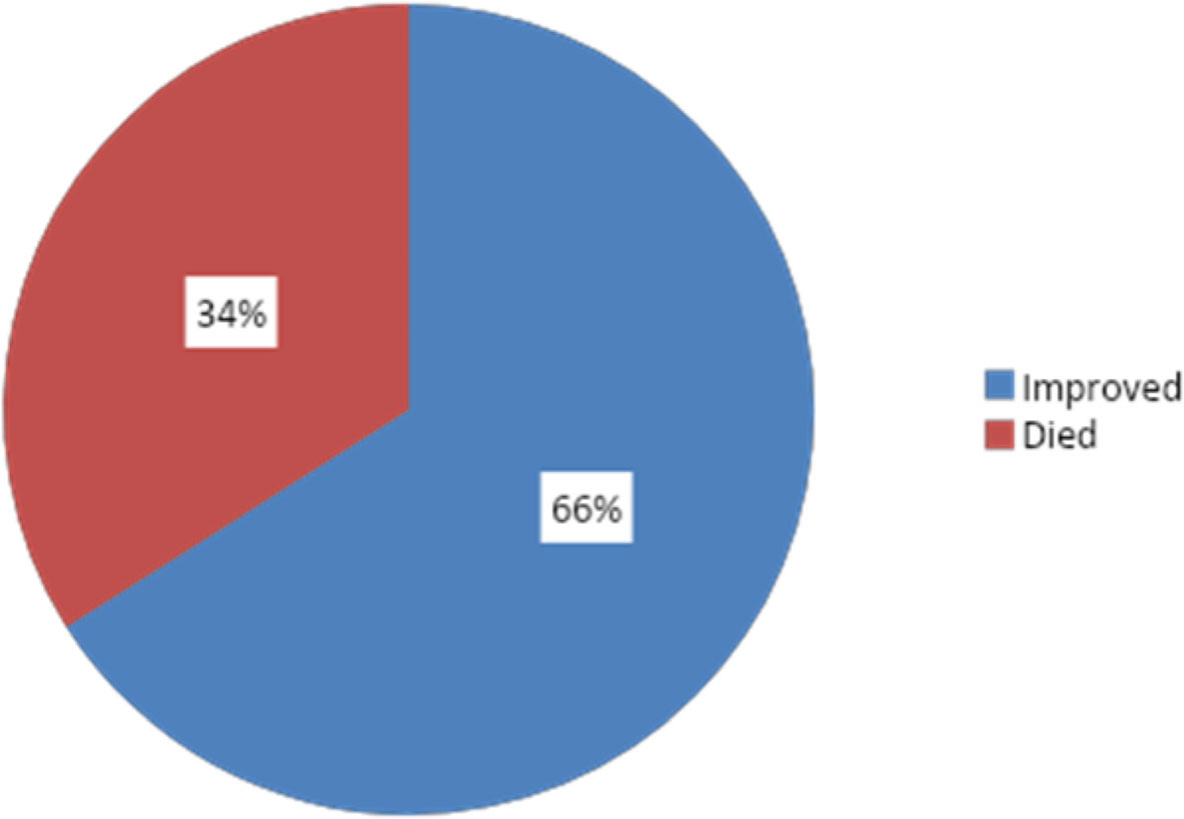
Outcome of patients with CAP

The current study included 153 with community-acquired pneumonia (CAP). Out of them, 101 (66%) patients improved while 52 (34%) died as shown in Fig. 1.

The characteristic data of the enrolled patients are summarized in Table 1. Mean age of improved patients was 49.47 ± 10.96 years. Majority (67.3%) of them were males and 34 (33.7%) of them were smokers. Mean age of patients who died was 59.88 ± 13.08 years and 29 (55.8%) of them were females. It was noticed that 18 (34.6%) patients who died were smokers. It was noticed that 20 (19.8%) patients who improved and 26 (30.8%) patients who died had co-existing comorbidities with significant differences between both groups. It was noticed that class of PSI was II, III, IV, and V in 5 (5%), 12 (11.9%), 30 (29.7%), and 54 (53.5%), respectively, of improved patients and in 5 (9.6%), 12 (23.1%), 14 (26.9%), and 21 (40.4%) of patients died, respectively. Mean PSI was significantly higher in patients who died (134.92 ± 51.84, vs.123.39 ± 42.62 *P* = 0.01). Moreover, CURB-65 was significantly higher in patients who died in comparison to improved patients (3.96 ± 1.13 vs. 3.33 ± 0.92; *P* < 0.001).

**Table 1:** Baseline characteristics of both groups

	Improved (*n* = 101)	Died (*n* = 52)	*P* value
Age (year)	49.47 ± 10.96	59.88 ± 13.08	0.01
Sex			0.01
Male	68 (67.3%)	23 (44.2%)	
Female	33 (32.7%)	29 (55.8%)	
Smoking			0.92
Current smoking	34 (33.7%)	18 (34.6%)	
None	40 (39.6%)	22 (42.3%)	
Stopped smoker	18 (17.8%)	9 (17.3%)	
Ex-smokers	9 (8.9%)	3 (5.8%)	
Comorbid diseases	20 (19.8%)	16 (30.8%)	0.04
CURB-65	3.33 ± 0.92	3.96 ± 1.13	< 0.001
PSI	123.39 ± 42.62	134.92 ± 51.84	0.01
class			0.01
II	15 (15%)	5 (9.6%)	
III	32 (31.7%)	12 (23.1%)	
IV	30 (29.7%)	14 (26.9%)	
V	24 (23.6%)	21 (40.4%)	
Laboratory data			
RDW (%)	15.50 ± 2.81	16.50 ± 3.54	0.02
Delta MPV	1.78 ± 0.76	2.61 ± 1.01	0.01
WBCs (× 10^9^/l)	13.80 ± 6.51	13.91 ± 7.93	0.92
Platelets (× 10^9^/l)	269.40 ± 54.87	269.90 ± 99.93	0.98
PaO_2_ /Fio_2_	166.37 ± 62.27	182.96 ± 64.68	0.12
Hospital stay (day)	12.97 ± 5.67	14.25 ± 6.80	0.22
Need to MV	38 (37.6%)	21 (40.4%)	0.68
Transfer to ICU	51 (50.5%)	31 (59.6%)	0.18
Radiological findings			0.45
Unilobar pneumonia	68 (67.3)	32 (61.5%)	
Multilobar pneumonia	33 (32.7%)	20 (38.5%)	
Effusion	17 (16.8%)	11 (21.2%)	
Positive blood culture	2 (2%)	6 (11.5%)	0.07
Positive sputum culture	95 (94.1%)	46 (88.5%)	0.46

Data expressed as mean (SD), frequency (percentage). *P* value was significant if < 0.05. *MV* mechanical ventilation, *ICU* intensive care unit

Delta MPV and RDW was significantly higher in patients who died (2.61 ± 1.01 vs. 1.78 ± 0.76; *P* = 0.01 for delta MPV and 16.50 ± 3.54 vs. 15.50 ± 2.81; *P* = 0.02 for RDW). Our research also detected that comorbid diseases, transfer to ICU, and need for mechanical ventilation were highly frequent in patients who died. Moreover, patients who died had longer duration of hospital stay. On radiological findings, pleural effusion and unilobar pneumonia were presented in 21.2% and 61.5% of patients who died vs. 16.8% and 67.3% of improved patients, respectively, while multilobar pneumonia was more frequent in patients who died. Blood culture was positive only in 2% of patients who improved vs. 11.5% of patients who died.

**Fig. 2:**
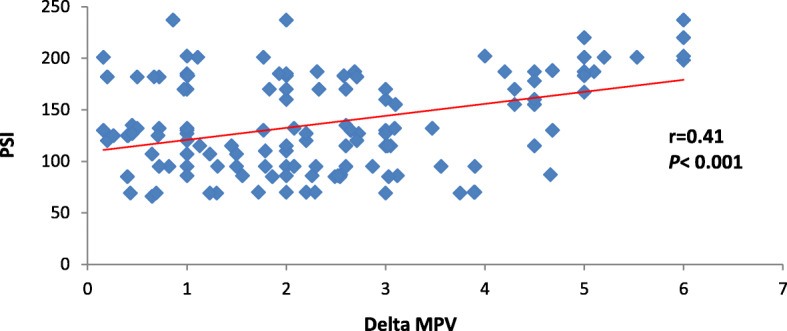
Correlation between delta MPV and PSI

**Fig. 3:**
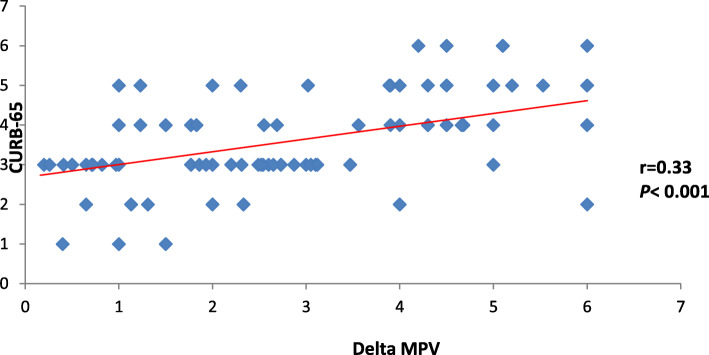
Correlation between delta MPV and CURB-65

**Fig. 4:**
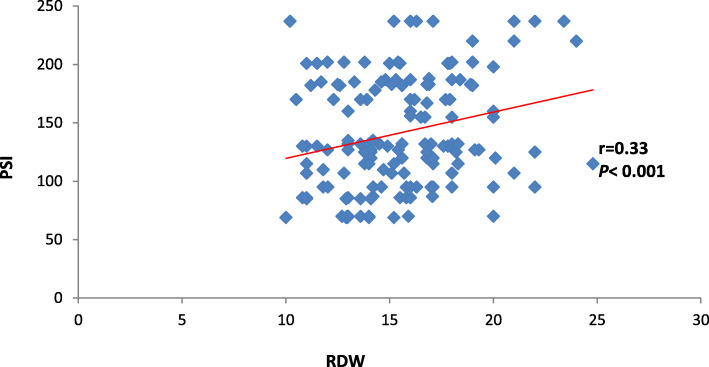
Correlation between RDW and PSI

**Fig. 5:**
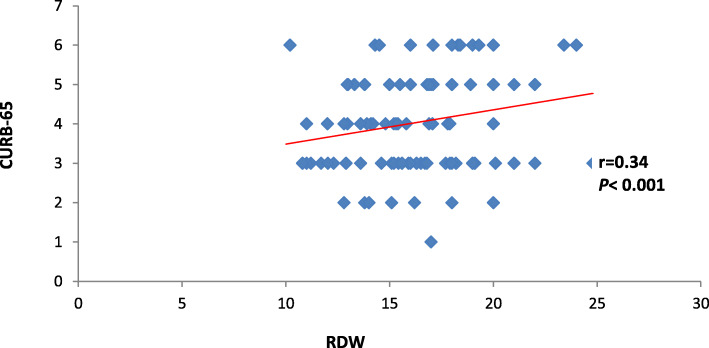
Correlation between RDW and CURB-65

The current study also discovered that each of ∆ MPV and RDW had positive significant correlation with PSI and CURB-65 (*P* < 0.001) as shown in Table 2 and Figs. 2, 3, 4, and 5.

**Table 2:** Correlation between CURB-65 and PSI in correlation to delta MPV and RDW

	Delta MPV		RDW	
	*r*	*P*	*r*	*P*
PSI	0.41	< 0.001	0.33	< 0.001
CURB-65	0.39	< 0.001	0.34	< 0.001

Date expressed as *r* (strength of correlation), *P* (significance of correlation). *P* value was significant if < 0.05. *MPV* mean platelet volume, *RDW* red cell distribution width, *PSI* pneumonia severity index

Based on the current study (Table 3), the following variables were predictors of in-hospital mortality in patients with CAP with adjusted *R*^2^ was 0.65; CURB-65 (OR = 1.46, 95% CI = 1.33–4.54; *P* = 0.03). PSI (OR = 1.99, 95% CI = 1.98–6.67; *P* = 0.03). RDW (OR = 1.22, 95% CI = 1.10–1.47; *P* = 0.02). Delta MPV (OR = 3.36, 95% CI = 2.22–7.66; *P* = 0.01).

**Table 3:** Predictors of in-hospital mortality in patients with CAP

	Odd’s ratio	95% confidence interval	*P* value
Age	0.99	0.95–1.04	0.24
Sex	0.33	0.12–0.89	0.09
Comorbidities	1.59	0.34–7.48	0.45
CURB-65	1.46	1.33–4.54	0.03
PSI	1.99	1.98–6.67	0.03
RDW	1.22	1.10–1.47	0.02
Delta MPV	3.36	2.22–7.66	0.01

*P* value was significant if < 0.05. *MPV* mean platelet volume, *RDW* red cell distribution width, *PSI* pneumonia severity index, *CAP* community-acquired pneumonia

**Fig. 6:**
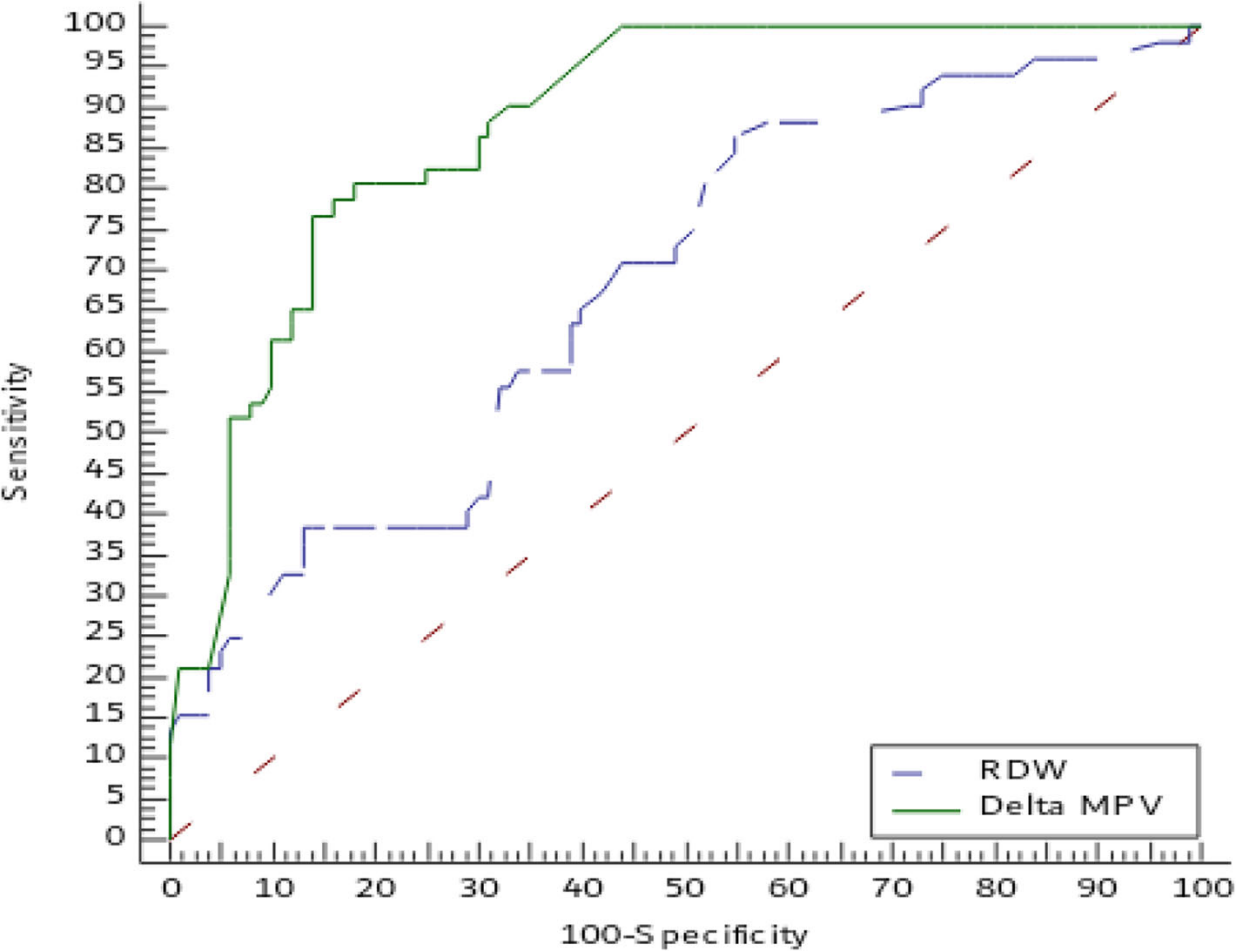
Diagnostic performance of delta MPV and RDW in prediction of in-hospital mortality

It was worthwhile to notice that RDW at cut-off point > 17.7% had 39% sensitivity and 87% specificity for prediction of mortality in patient with CAP with area under curve 0.68 while delta MPV at cut-off point > 2.92% had 77% sensitivity and 86% specificity for prediction of death in patient with CAP with area under curve 0.87 as shown in Table 4 and Fig. 6.

**Table 4:** Performance of RDW and delta MPV in prediction of mortality in CAP

	RDW	Delta MPV
Sensitivity	39%	77%
Specificity	87%	86%
Positive predictive value	61%	74%
Negative predictive value	73.3%	88%
Cut-off point	> 17.7	> 2.92
Area under curve	0.68	0.87
*P* value	< 0.001	< 0.001

*P* value was significant if < 0.05. *RDW* red cell distribution width, *MPV* mean platelet volume, *CAP* community-acquired pneumonia

## Discussion

In the recent few years, CAP had been considered one of the leading causes of death worldwide. Therefore, augmentation of the conventional severity scores, like the PSI and CURB-65, became a must to identify patients with high risk for a complex course as the predictive performance of these scores alone may be limited. Several researches have detected that discovering new biomarkers could augment the validity of these severity scores [21, 24].

In this prospective study, we planned to assess the validity of the mean platelet volume change and RDW as biomarkers for assessing the severity of CAP. It was worthwhile to know that no previous study has been done at Assiut University Hospital to assess those two biomarkers in patients with CAP. The main potential mechanism for rising MPV in patient population is severe inflammation caused by pneumonia. In severe infection, increased release of thrombopoietin and various inflammatory cytokines, such as interleukin-1, -3 and -6, and tumor necrosis factor-α, result in increased thrombopoiesis and enhanced expression of younger large platelets into the blood circulation [7, 25–27]. On the other hand, rising MPV may be attributed to increased thrombocyte consumption in the peripheral tissue and spleen, induced by severe inflammatory status [7, 26, 27]. Community-acquired pneumonia is an infectious disease that results in inflammatory and oxidative stress to the host. If these stresses are severe, mortality will be increased. The underlying pathophysiological mechanisms for a relationship of rising MPV with poor prognosis and mortality are not fully understood. The main potential explanation can be increased platelet activation [27, 28]. Larger thrombocytes are known to be functionally, metabolically and enzymatically more active than smaller ones. The greater activation of enlarged platelets results in increased release of procoagulant substances such as thromboxane A2, β-thromboglobulin, and surface proteins [27, 28]. Consequent hyperaggregability of platelets, extended vasoconstriction, and endothelial dysfunction may contribute to an increased short-term risk of cardiovascular thrombosis and death in patients with rising MPV [26, 27].

Our research detected that a high RDW and rising MPV were significantly related with increased risk of death in patients with CAP as delta MPV and RDW was significantly higher in patients who died (2.61 ± 1.01 vs. 1.78 ± 0.76; *P* = 0.01) for delta MPV and (16.50 ± 3.54 vs. 15.50 ± 2.81; *P* = 0.02) for RDW. Our results are in agreement with Braun et al. 2011 [22] who detected that RDW was associated with high risk of death and disability in young patients admitted with CAP. In this retrospective study, Brawn et al., in a cohort of patients of 60 years or older hospitalized due to CAP, demonstrated that elevated RDW (> 14.5%) was independently associated with complicated hospitalization (length of stay > 10 days and admission to ICU) and 90-day mortality, irrespective of hemoglobin levels [22]. In line with the results of our study, Lee et al. also identified a high RDW is a prognostic factor for 30-day CAP mortality [21]. Age is significant prognostic factor in various diseases, including CAP. In our study, the mean age of improved patients with CAP was 49.47 ± 10.96 years, while mean age of patients who died was 59.88 ± 13.08 years. Thus, our findings support RDW as a significant prognostic factor in patients with CAP across all ages; these results are in agreement with Braun et al. 201 1[22]. Their finding is similar to our results; however, they only included patients who were younger than 60 years, which they cited as a limitation of their study. This study revealed that each of PSI and CURB-65 had positive significant correlation with delta MPV and RDW (*P* < 0.001). Our results are also in line with Gorelik et al. 2017 who stated that rising MPV during hospitalization for CAP is associated with a more severe clinical profile and mortality than no rise in MPV. They found that MPV values above the cut-off at discharge were associated with an increased risk of mechanical ventilation and death during hospitalization, and shortened survival following discharge [29].

Based on the current study, the following variables were predictors of in-hospital mortality in patients with CAP with adjusted *R*^2^ was 0.65; CURB-65 (OR = 1.46, 95% CI = 1.33–4.54; *P* = 0.03), PSI (OR = 1.99, 95% CI = 1.98–6.67; *P* = 0.03), RDW (OR = 1.22, 95% CI = 1.10–1.47; *P* = 0.02). Delta MPV (OR = 3.36, 95% CI = 2.22–7.66; *P* = 0.01). Indeed, in our patient population, a rise in MPV was associated with higher pneumonia severity scores.

In our study, the mortality prediction of both the PSI and CURB-65 was improved by the addition of RDW and delta MPV as severity criteria. The exact mechanisms of an association of RDW with the mortality of CAP need to be determined. It has been suggested that inflammation and oxidative stress affect red cell homeostasis. A previous study revealed that RDW displayed a strong, graded association with inflammatory biomarkers in general outpatient populations [30], and another study indicated that serum antioxidant levels including selenium and carotenoids were associated with RDW in older women [31]. In our data, patients with a higher RDW had a tendency toward higher severity index scores, and the overall mortality was also higher in patients with a higher RDW.

There are some limitations of our research work. First, the study included one group of CAP patients who were admitted in our Assiut Chest Department and RICU. Thus, it cannot be generalized to all patients with CAP. Second, larger studies of large numbers of patients need to be done to investigate the value of mean platelet volume change and RDW as prognostic markers in community-acquired pneumonia.

## Conclusions

RDW and rising MPV during hospitalization for CAP is associated with more severe clinical characteristics and high mortality. We suggest that repeated MPV and RDW determination throughout hospitalization may improve risk stratification for CAP patients.

## Data Availability

Data and materials are available.

## Abbreviations

CAP: Community-acquired pneumonia
CBC: Complete blood count
CRB-65: Confusion, respiratory rate, blood pressure, age ≥ 65
CURB-65: Confusion, urea, respiratory rate, blood pressure, age ≥ 65
∆MPV: Mean platelet volume change
ED: Emergency department
ICU: Intensive care unit
RDW: Blood cell distribution width
